# Return to work and health-related quality of life up to 1 year in patients hospitalized for COVID-19: the CO-FLOW study

**DOI:** 10.1186/s12916-023-03083-3

**Published:** 2023-10-02

**Authors:** L. M. Bek, J. C. Berentschot, M. E. Hellemons, S. C. Remerie, J. van Bommel, J. G. J. V. Aerts, G. M. Ribbers, H. J. G. van den Berg-Emons, M. H. Heijenbrok-Kal, on behalf of the CO-FLOW collaboration group, Michel E. van Genderen, Diederik A. M. P. J. Gommers, Erwin Ista, Robert van der Stoep, Rutger Osterthun, Markus P. J. M. Wijffels, Marieke M. Visser, Janette J. Tazmi-Staal, Eva G. Willems, Roxane Heller, Shai A. Gajadin, Wouter J. B. Blox, Laurien Oswald, Sieshem Bindraban, Herbert J. van de Sande, Ronald N. van Rossem, Stephanie van Loon-Kooij

**Affiliations:** 1 Department of Rehabilitation Medicine, Erasmus MC, University Medical Center Rotterdam, Rotterdam, The Netherlands; 2 Department of Respiratory Medicine, Erasmus MC, University Medical Center Rotterdam, Rotterdam, The Netherlands; 3 Rijndam Rehabilitation, Rotterdam, The Netherlands; 4 Department of Adult Intensive Care Medicine, Erasmus MC, University Medical Center Rotterdam, Rotterdam, The Netherlands

**Keywords:** Return to work, Quality of life, COVID-19, Intensive care, Fatigue, Rehabilitation, Mental health

## Abstract

**Background:**

Currently, evidence about the long-term consequences of COVID-19 on return to work and health-related quality of life (HRQoL) is limited. We evaluated return to work and its associations with baseline characteristics and physical and mental recovery over time in patients up to 1 year after hospitalization for COVID-19. Secondly, we aimed to evaluate the association between return to work and health-related quality of life (HRQoL).

**Methods:**

CO-FLOW, a multicenter prospective cohort study, enrolled adult participants hospitalized for COVID-19, aged ≥ 18 years within 6 months after hospital discharge. Return to work and HRQoL were collected at 3, 6, and 12 months after hospital discharge using the iMTA Productivity Cost Questionnaire and the 36-Item Short Form Health Survey, respectively. Data were collected between July 1, 2020, and September 1, 2022. Generalized estimating equations with repeated measurements were used to assess outcomes over time.

**Results:**

In the CO-FLOW study, 371 participants were employed pre-hospitalization. At 3, 6, and 12 months post-discharge, 50% (170/342), 29% (92/317), and 15% (44/295) of participants had not returned to work, and 21% (71/342), 21% (65/317), and 16% (48/295) only partially, respectively. ICU admission (adjusted odds ratio (95% confidence interval): 0.17 (0.10 to 0.30), *p* < 0.001), persistent fatigue (0.93 (0.90 to 0.97), *p* < 0.001), female sex (0.57 (0.36 to 0.90), *p* = 0.017), and older age (0.96 (0.93 to 0.98), *p* < 0.001) were independently associated with no return to work. ICU patients required a longer time to return to work than non-ICU patients. Patients who did not return or partially returned to work reported lower scores on all domains of HRQoL than those who fully returned.

**Conclusions:**

One year after hospitalization for COVID-19, only 69% of patients fully returned to work, whereas 15% did not return and 16% partially returned to work. No or partial return to work was associated with reduced HRQoL. This study suggests that long-term vocational support might be needed to facilitate return to work.

**Trial registration:**

World Health Organization International Clinical Trials Registry Platform NL8710.

**Supplementary Information:**

The online version contains supplementary material available at 10.1186/s12916-023-03083-3.

## Background

More than 3 years after the outbreak of coronavirus disease 2019 (COVID-19), reports show that up to 90% of the patients hospitalized for COVID-19 experience a wide range of physical, cognitive, and mental health consequences up to 1 year after COVID-19 [1–4]. These consequences could largely impact patients’ participation in society, including return to work. While studies have shown long-lasting problems with return to work, the proportion of patients not able to return to work varies widely, ranging from 6 to 43% at 6 months and 11 to 88% at 1 year after hospitalization for COVID-19 [4–8].

A small cohort study showed that patients admitted to the intensive care unit (ICU) report a four times longer time to return to work compared with those treated at the general ward at 7 months after hospital discharge [9]. However, the longitudinal pattern of return to work and other factors beyond ICU admission that may impact the ability to return to work are currently poorly understood.

Long-term sick leave not only impacts society’s productivity costs, but also largely affects patients and their families in their social life, finances, and overall well-being [10, 11]. Up to 1 year post-COVID-19, patients report a reduced HRQoL [12]. Small cohort studies showed that patients who did not return to work reported a worse HRQoL than those who did [7, 13]. Moreover, ICU admission, female sex, and presence of post-COVID-19 symptoms, including fatigue, depression, and muscle weakness, are associated with a reduced HRQoL [14–16].

The current literature lacks large prospective cohort studies that repeatedly evaluate return to work over time, its risk factors, and its association with HRQoL up to 1 year post-COVID-19. However, as numerous patients experience long-term problems, understanding these aspects is crucial to optimize treatment. We sought to investigate the following aims within the CO-FLOW study, a large multicenter cohort study (*N* = 650) following patients hospitalized for COVID-19 up to 2 years after hospital discharge with a large proportion of patients who have been admitted to the ICU. First, we aimed to evaluate return to work over time and its associations with baseline characteristics and physical and mental recovery over time in patients up to 1 year after hospitalization for COVID-19. Second, we evaluated the association between return to work and HRQoL. We hypothesized that return to work improves over time, but takes longer for patients admitted to the ICU compared with those who are not. Second, we expected that patients unable to return to work experience a worse HRQoL.

## Methods

### Study design and participants

Participants of the COvid-19 Follow-up care paths and Long-term Outcomes Within the Dutch healthcare system (CO-FLOW) study who were employed prior to hospitalization for COVID-19 were selected for this study. The design of the CO-FLOW study has been described previously [17]. In short, individuals hospitalized because of COVID-19 are followed up at 3, 6, 12, and 24 months after discharge within the southwest of the Netherlands. The current analysis focuses on measurements up to 12 months. Data were collected between July 1, 2020, and September 1, 2022. We included adult patients (≥ 18 years) with a confirmed COVID-19 diagnosis within 6 months after hospital discharge who were fluent in Dutch or English. Incapacitated patients were not included because of the study procedure. All participants provided written informed consent. The Medical Ethics Committee of the Erasmus Medical Center approved this study (MEC-2020–0487). The study is registered on the World Health Organization International Clinical Trials Registry Platform (NL8710) and reported in accordance with the STROBE guidelines [18].

### Measurements

Personal and clinical characteristics were obtained at study visits at 3, 6, and 12 months and retrospectively from electronic patient records. Alongside each study visit, patient-reported outcome measures (PROMs) were collected using standardized questionnaires via email or postal mail [17].

#### Primary outcome

Return to work was evaluated with the iMTA Productivity Cost Questionnaire (iPCQ) [19]. The iPCQ enquires information regarding occupation, paid or unpaid work, number of workdays, hours per week of paid work, and short-term (≤ 4 weeks) and long-term (> 4 weeks) absence from paid work. Occupation was categorized into 3 categories: (1) white collar, including executive, administrative, and managerial (technical) occupations; (2) manual labor; and (3) service, including healthcare support, education, protective service, and personal care occupations [20]. In case iPCQ was missing, return to work was evaluated via the first item of the Utrecht Scale for Evaluation of Rehabilitation—Participation that asks hours per week of paid work [21]. If both were missing, information regarding return to work was collected via a telephone interview.

#### Secondary outcome

HRQoL was assessed with the 36-Item Short Form Health Survey (SF-36). The SF-36 contains eight domains: physical functioning (PF), role limitations due to physical health (RP), role limitations due to emotional problems (RE), vitality (VT), mental health (MH), social functioning (SF), bodily pain (BP), and general health (GH) [22, 23]. Each domain is transformed to a scale ranging from 0 (worst health) to 100 (best health). Two higher-order summary scores are calculated: Physical Component Summary (PCS) by positively weighing the PF, RP, BP, VT, and GH, and negatively weighing RE, MH, and SF; and the Mental Component Summary (MCS) by positively weighing RE, VT, MH, and SF, and negatively weighing PF, RP, BP, and GH [24]. The PCS and MCS are *T*-scores, having a normal mean score of 50 and SD of 10. The SF-36 has been extensively validated in the Dutch population [22].

#### Baseline characteristics and recovery status

Personal characteristics included age at admission, sex, body mass index (BMI) at admission, migration background (European/non-European), and pre-hospitalization educational (low [primary or secondary education]; middle [high school]; high [postsecondary education or university]) status. Clinical characteristics included comorbidities, length of stay (LOS) in hospital in days, type of COVID-19-directed treatment during hospitalization (no treatment/steroids/antivirals/anti-inflammatory/(hydroxy)chloroquine/convalescent plasma), oxygen support, ICU admission, and LOS in ICU in days.

Recovery status was assessed at 3, 6, and 12 months after hospitalization. We assessed physical fitness by the 6-minute walk test (6MWT) and 1-minute sit-to-stand test (1MSTST) to assess cardiorespiratory fitness, and maximum isometric handgrip strength (HGS) to assess overall muscle strength, resulting in the following outcomes that were all normalized to percentage of normative values (%pred): 6-minute walking distance in meters (6MWD) [25], number of sit-to-stand (STS) repetitions [26], and maximum HGS in kg [27]. At 3, 6, and 12 months follow-up, we also collected PROMs on cognitive failures (Cognitive Failures Questionnaire, scoring range 0–100 [28]), fatigue (Fatigue Assessment Scale, scoring range 10–50 [29]), anxiety and depression (Hospital Anxiety and Depression Scale; Anxiety subscale and Depression subscale, scoring range 0–21 [30]), and posttraumatic stress disorder (PTSD) (Impact of Event Scale-Revised, scoring range 0–88 [31]).

### Data analysis

Continuous variables are presented as mean (SD) and/or median (IQR); categorical variables as n (%). Descriptive statistics were used to check statistical assumptions. Generalized estimating equations (GEE) analyses with repeated measurements for binary logistic models using an unstructured working correlation matrix were performed to evaluate return to work over time and to identify variables associated with return to work via univariable and subsequent multivariable analyses. We categorized return to work into no, partial, i.e., working less hours than pre-hospitalization due to COVID consequences, or full, i.e., equal hours as pre-hospitalization. Primary analysis concerned patients who did not return to work versus those who partially or fully returned to work. Secondary analysis included patients who did not return or partially returned to work versus those who fully returned. In addition, we used GEE for linear models with repeated measurements to evaluate HRQoL over time and the independent association between return to work and HRQoL adjusted for covariables. In the univariable models, time together with the most prevalent comorbidities (> 20%, history of obesity, cardiovascular disease, and pulmonary disease), ICU admission, and LOS hospital, and the time-varying variables occupational category, 6MWD %pred, STS %pred, HGS %pred, cognitive failures, fatigue, anxiety, depression, and PTSD were entered. To assess whether ICU patients required longer time to return to work than non-ICU patients, we entered an interaction term for ICU admission with follow-up time. Significant variables (*p* < 0.05) were entered into the multivariable models, however, if multicollinearity (Spearman’s rho ≥ 0.6) was present, ICU was preferred over LOS hospital, depression over anxiety and PTSD, and STS %pred over 6MWD %pred in the multivariable models, based on our aim, literature, and univariable significance level [2, 32]. Little’s missing completely at random test was performed for variables with missing values (BMI at admission, occupational category, 6MWT, HGS, STS, fatigue, cognitive failures, anxiety, depression, and PTSD) showing patterns in missing data (*p* ≤ 0.001). We addressed missing data with multiple imputation under the missing-at-random assumption (one hundred datasets, hundred iterations, predictive mean matching (*K* = 5), final models aggregated using Rubin’s Rules). Model parameters are presented by forest plots showing the estimated adjusted odds ratios (AOR) for return to work or estimated mean differences for PCS and MCS with corresponding 95% confidence intervals (CI), and *p*-values, using *p* < 0.05 as significance level. Statistical analyses were carried out using SPSS version 28 (IBM SPSS Statistics, SPSS Inc., Chicago, IL, USA) and R version 4.2.1 (R-Foundation) were used for graphs.

## Results

### Study population

**Fig. 1:**
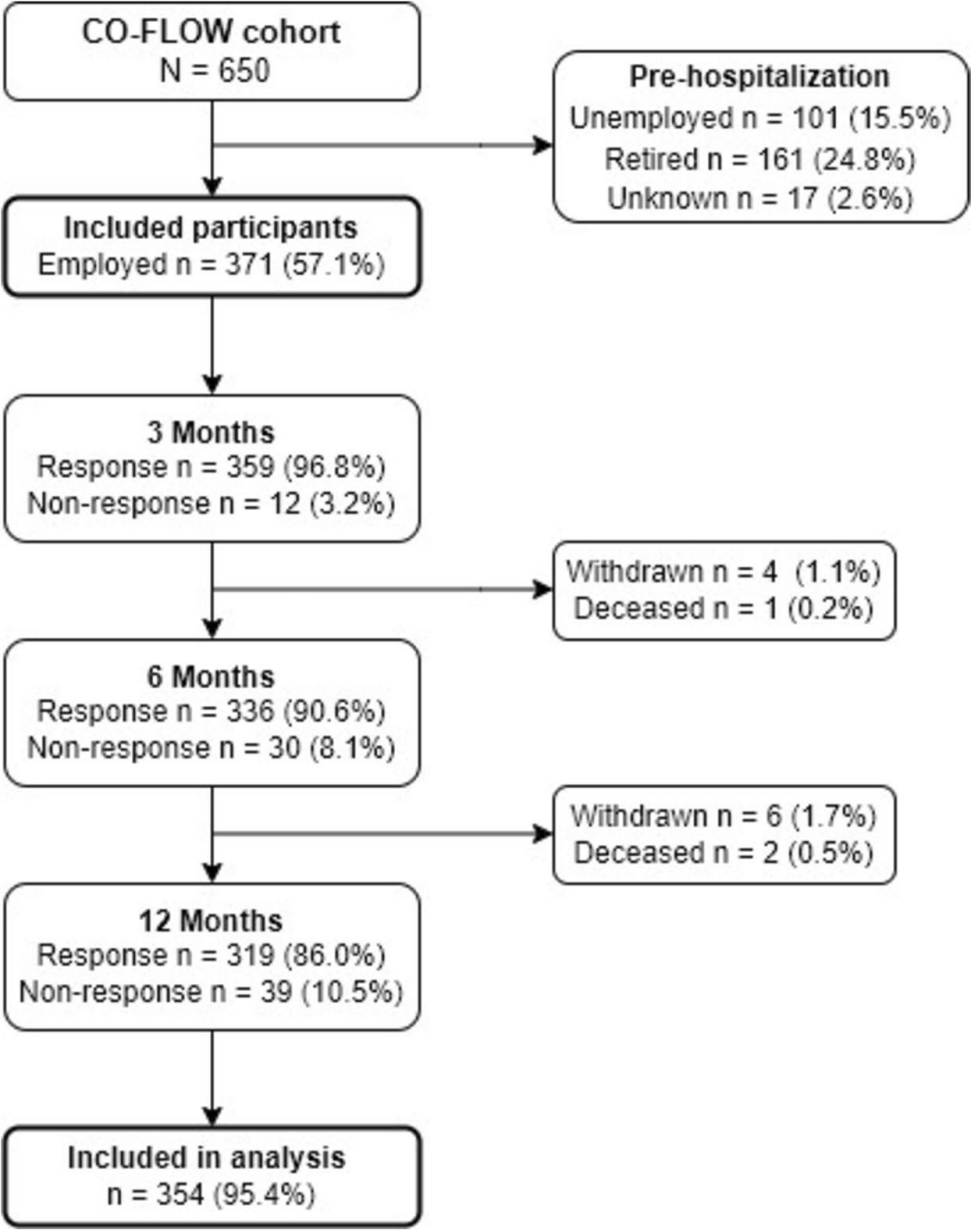
Flowchart of inclusion procedure for the study sample. Of the 650 patients, 371 were employed prior to hospitalization for COVID-19 and were included in the study sample. The final generalized estimating equations analyses with full model specification included 354 (95.4%) patients

Of the 650 patients enrolled in the CO-FLOW study, 371/650 (57.1%) patients were employed pre-hospitalization and included in the study sample (Fig. 1). Of these, 359 (96.8%), 336 (90.6%), and 319 (86.0%) patients responded at 3, 6, and 12 months, respectively. At 12 months, responders did not differ significantly from non-responders regarding sex and LOS hospital (*p* = 0.3; *p* = 0.5); however, responders were significantly older (55.7 [8.8] vs 48.4 [10.6], *p* < 0.001) and less frequently admitted to the ICU (41.4% vs 61.5%, *p* = 0.017). At hospital admission, the median age was 57.0 (50.0–61.0) years, median BMI was 28.4 (25.8–32.2), 104 (28.0%) patients were female, and 295/371 (79.5%) patients had ≥ 1 comorbidities (Table 1). Median LOS in the hospital was 11.0 (6.0–29.0) days and 161 (43.4%) patients were admitted to the ICU with a median LOS in ICU of 18.0 (9.0–31.0) days.

**Table 1:** Demographics and clinical characteristics of the study population

	Study population (*N* = 371)
**Demographics**	
Age at admission, *years*	
Mean (SD)	55.0 (9.4)
Median (IQR)	57.0 (50.0–61.0)
Sex, *female*	104 (28.0)
BMI at admission, *kg/m*^*2*a^	
Mean (SD)	29.5 (5.3)
Median (IQR)	28.4 (25.8–32.2)
*Migration background*	
European	258 (69.5)
Dutch Caribbean	56 (15.1)
Asian	24 (6.5)
Turkish	17 (4.6)
(North) African	16 (1.3)
*Educational level*	
Low	106 (28.6)
Middle	137 (36.9)
High	128 (34.5)
**Clinical characteristics**	
*Comorbidities*	
≥ 1	295 (79.5)
Obesity (BMI ≥ 30)	160 (43.1)
Cardiovascular disease	122 (32.9)
Pulmonary disease	81 (21.8)
Diabetes	71 (19.1)
Autoimmune and/or inflammatory disease	33 (8.9)
Malignancy	29 (7.8)
Neurological disease	28 (7.5)
Renal disease	25 (6.7)
Gastrointestinal disease	23 (6.2)
Mental disorder	16 (4.3)
*Treatment *^*a*^	
No treatment	86 (23.2)
Steroids	259 (69.8)
Antivirals	54 (14.6)
Anti-inflammatory	50 (13.5)
(Hydroxy)chloroquine	9 (2.4)
Convalescent plasma	3 (0.8)
LOS hospital, *days*	
Mean (SD)	19.7 (20.0)
Median (IQR)	11.0 (6.0–29.0)
ICU admission	161 (43.4)
LOS ICU, *days *^*a*^	
Mean (SD)	21.8 (16.3)
Median (IQR)	18.0 (9.0–31.0)
Oxygen supplementation	362 (97.6)
High flow nasal cannula ^a^	122 (34.9)
Invasive mechanical ventilation	136 (36.7)
Length of intubation, *days *^*a*^	
Mean (SD)	20.3 (13.9)
Median (IQR)	16.0 (9.0–29.8)
Tracheostomy ^a^	56 (15.3)
**Time interval between hospital discharge and follow-up visit**	
3 months visit, *days*, mean (SD)	95.5 (13.9)
6 months visit, *days*, mean (SD)	187.0 (15.1)
12 months visit, *days*, mean (SD)	368.8 (14.7)

Data are presented as *n* (%), unless indicated. *BMI*, body mass index; *ICU*, intensive care unit; *LOS*, length of stay

^a^Missing values in BMI, *n* = 37; LOS in ICU, *n* = 1; treatment, *n* = 29, high flow nasal cannula, *n* = 21; length of intubation, *n* = 4; tracheostomy, *n* = 4

### Return to work

**Fig. 2:**
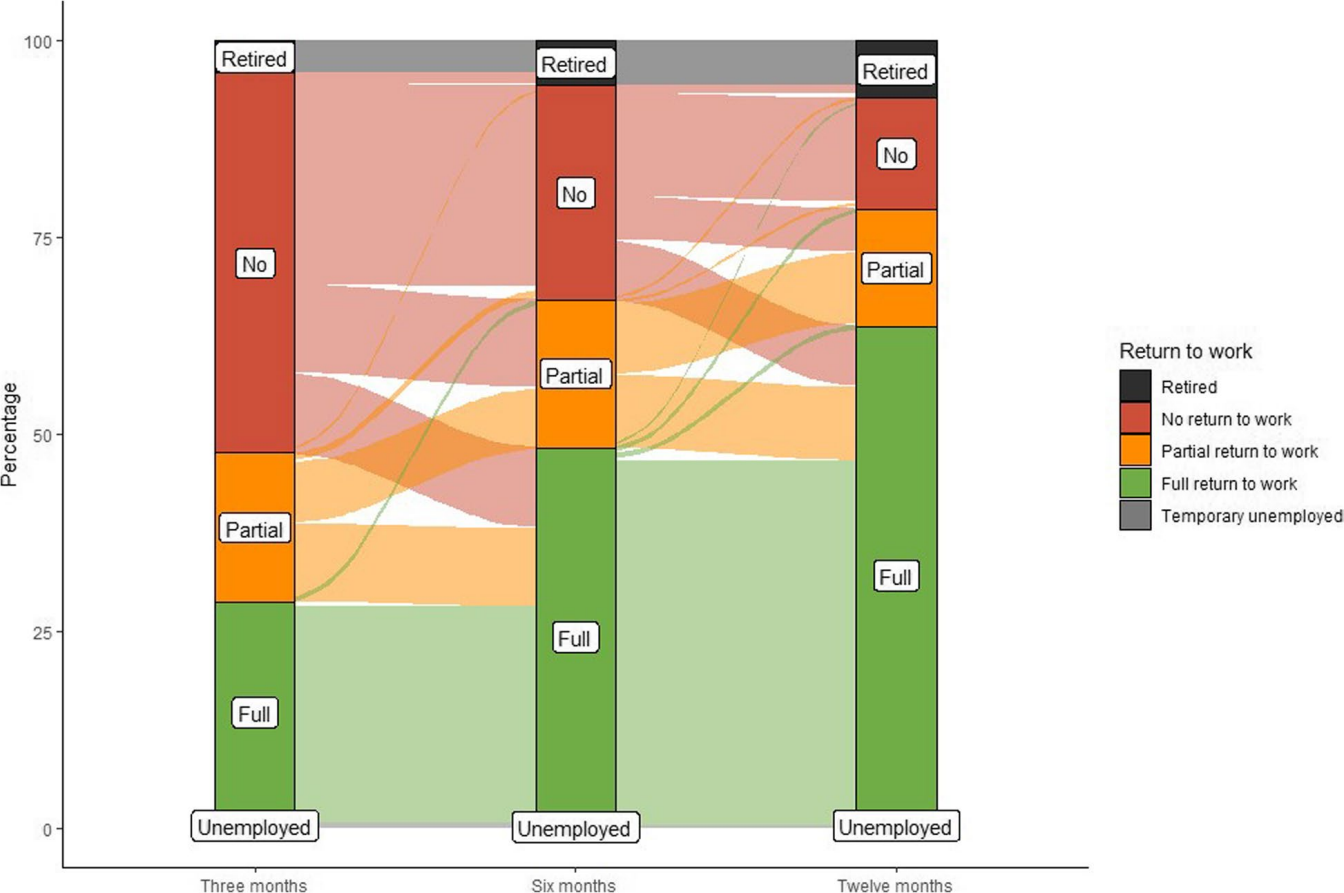
Alluvial plot showing changes in employment status (retired, no return to work, partial return to work, full return to work, or temporary unemployed) over the follow-up time of patients employed prior to hospitalization for COVID-19

**Fig. 3:**
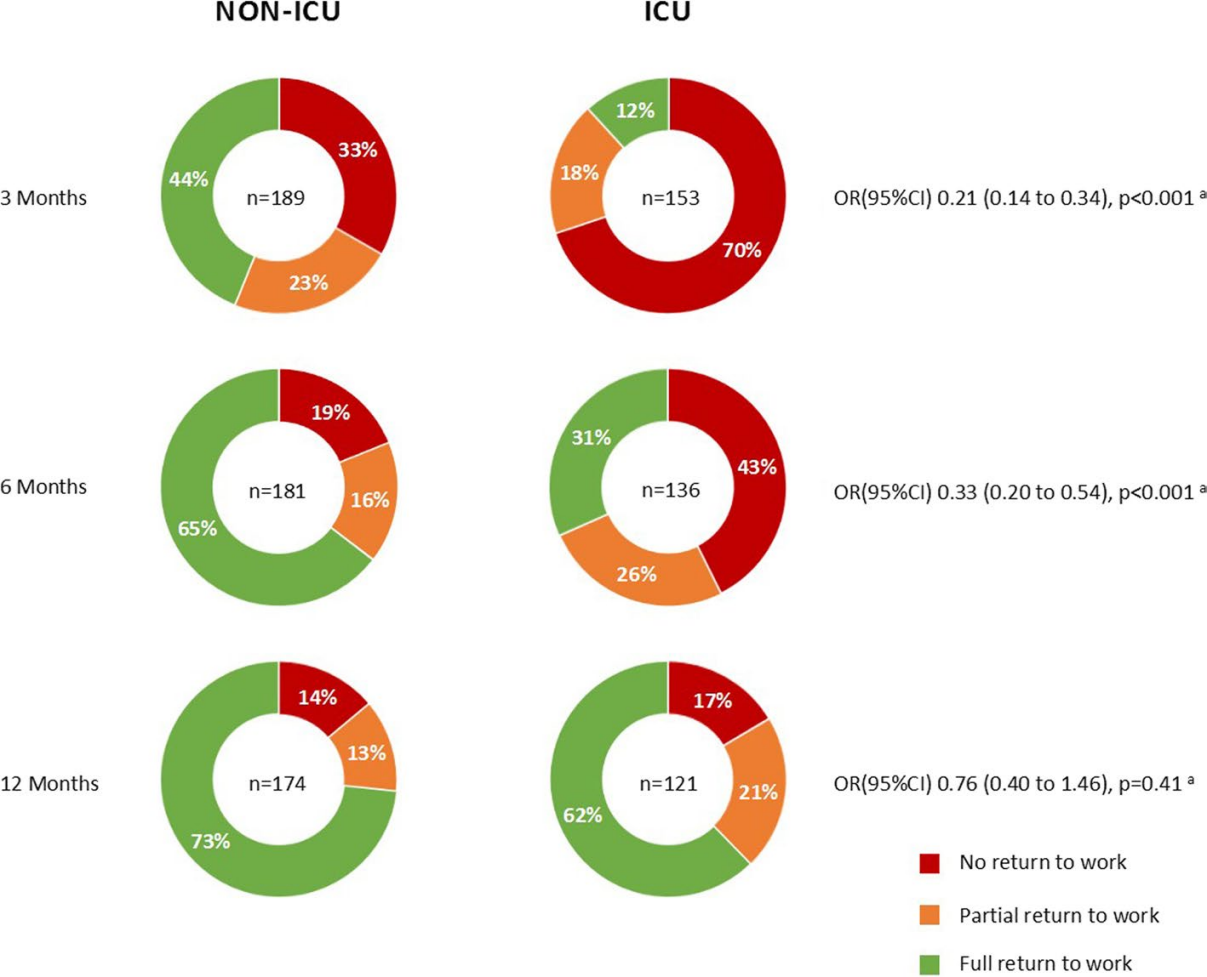
Plots presenting the percentage of non-ICU patients compared to ICU patients who did not return, partially returned, or fully returned to work at 3, 6, and 12 months after hospitalization for COVID-19. ICU, intensive care unit; OR, odds ratio; CI, confidence intervals. ^a^ Odds ratios are obtained from univariable Generalized Estimating Equations analysis with return to work (partial or full) as dependent variable

At 3 months follow-up, 49.7% (170/342) of the study population had not returned to work, which decreased to 29.0% (92/317) at 6 months and 14.9% (44/295) at 12 months. Moreover, 20.8% (71/342) of the participants only partially returned to work at 3 months, 20.5% (65/317) at 6 months, and 16.3% (48/295) at 12 months (Fig. 2). Overall, 68.8% (203/295) of the patients fully returned to work after 12 months. Comparing patients who had and those who had not been admitted to the ICU, we found that 30.1% (46/153) of the ICU patients at 3 months, 57.3% (78/136) at 6 months, and 83.6% (102/122) at 12 months returned to work against 66.7% (126/189), 81.2% (147/181), and 86.1% (149/173) of the non-ICU patients, respectively (Fig. 3).

Results of univariable analyses for return to work (Additional file 1: Fig. S1A) and full return to work (Additional file 1: Fig. S1B) are presented in the supplementary material.

**Fig. 4:**
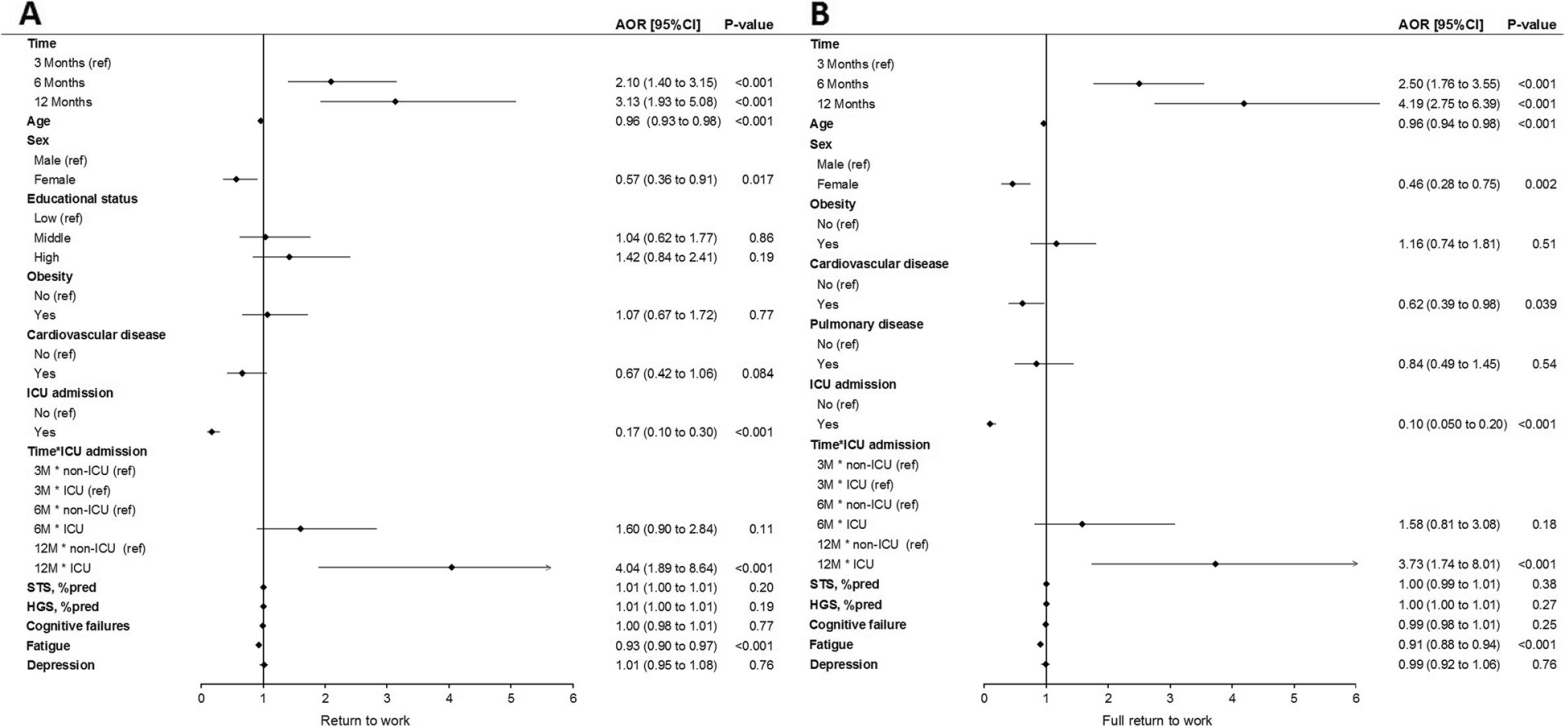
Forest plot showing adjusted odds ratios from multivariable analysis of **A** return to work (no versus partial/full) and **B** full return to work (no/partial versus full) up to 1 year after hospitalization for COVID-19. ICU, intensive care unit; M, months; STS, sit-to-stand; %pred, percentage of normative values; HGS, handgrip strength

The multivariable analysis showed that return to work significantly improved over time; patients were more likely to return to work at 6 months (OR (95% CI): 2.10 (1.40 to 3.15), *p* < 0.001) and 12 months (3.13 (1.93 to 5.08), *p* < 0.001) compared to 3 months after hospital discharge. ICU admission (0.17 (0.10 to 0.30), *p* < 0.001), age (0.96 (0.93 to 0.98), *p* < 0.001), sex (0.57 (0.36 to 0.91), *p* = 0.017), and fatigue (0.93 (0.90 to 0.97), *p* < 0.001) remained significantly associated with return to work (Fig. 4A). Overall, ICU patients were less likely to return to work (0.17 (0.10 to 0.30), *p* < 0.001) compared with non-ICU patients.

Also, ICU patients required more time to return to work, as they were less likely to return at 3 months (0.17 (0.10 to 0.30), *p* < 0.001), but changing from 3 to 12 months, their change in relative odds ratio of return to work (4.04 (1.89 to 8.64), *p* < 0.001)) was higher compared with non-ICU patients (Fig. 4A). This finding is also shown in Fig. 3. The same associations were found for full return to work along with a history of cardiovascular disease (Fig. 4B).

### Health-related quality of life

**Fig. 5:**
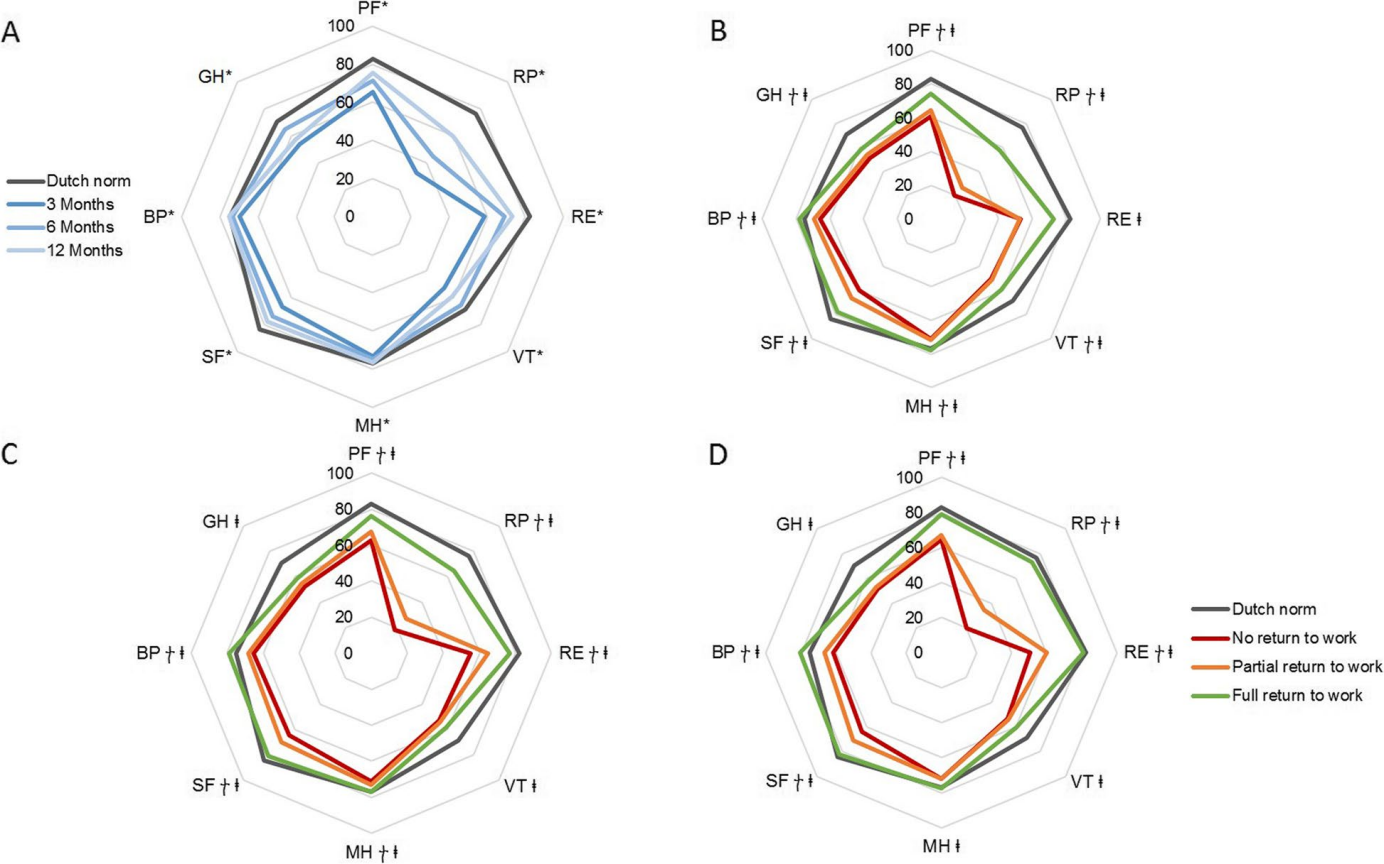
Radar plots representing HRQoL on the 8 domains of the SF-36 in the study population compared with the Dutch norm (gray line). **A** HRQoL on SF-36 domains for the total cohort at 3, 6, and 12 months. **B**–**D** HRQoL on SF-36 domains for participants with no (red line), partial (orange line), or full (green) return to work. In **B**, the results are shown for 3-months follow-up; in **C**, for 6-months follow-up; and in **D**, for 12-months follow-up. * Significant improvement over time (*p* < 0.007).  = significant difference in HRQoL between no return to work and partial/full return to work (*p* < 0.05).  = significant difference between no/partial return to work and full return to work (*p* < 0.03). PF, physical functioning; RP, role limitations due to physical health; RE, role limitations due to emotional problems; VT, vitality; MH, mental health; SF, social functioning; BP, bodily pain; GH, general health

All domains of the SF-36 improved over time (all domains, *p* < 0.007) (Fig. 5A, Additional file 1: Table S1 and S2). Most limitations were observed in RP, GH, VT, and RE at 1 year compared to the Dutch norm.

Patients who did not return to work reported significantly lower HRQoL on the SF-36 domains PH, RP, SF, BP, and PCS on all follow-up moments compared with patients who did partially or fully (*p* < 0.05) (Additional file 1: Table S3). Patients who did not return or partially returned to work reported significantly lower HRQoL on all SF-36 domains, and both PCS and MCS at 3, 6, and 12 months compared with those who fully returned (*p* < 0.03) (Fig. 5B–D, Additional file 1: Table S4 and S5).

**Fig. 6:**
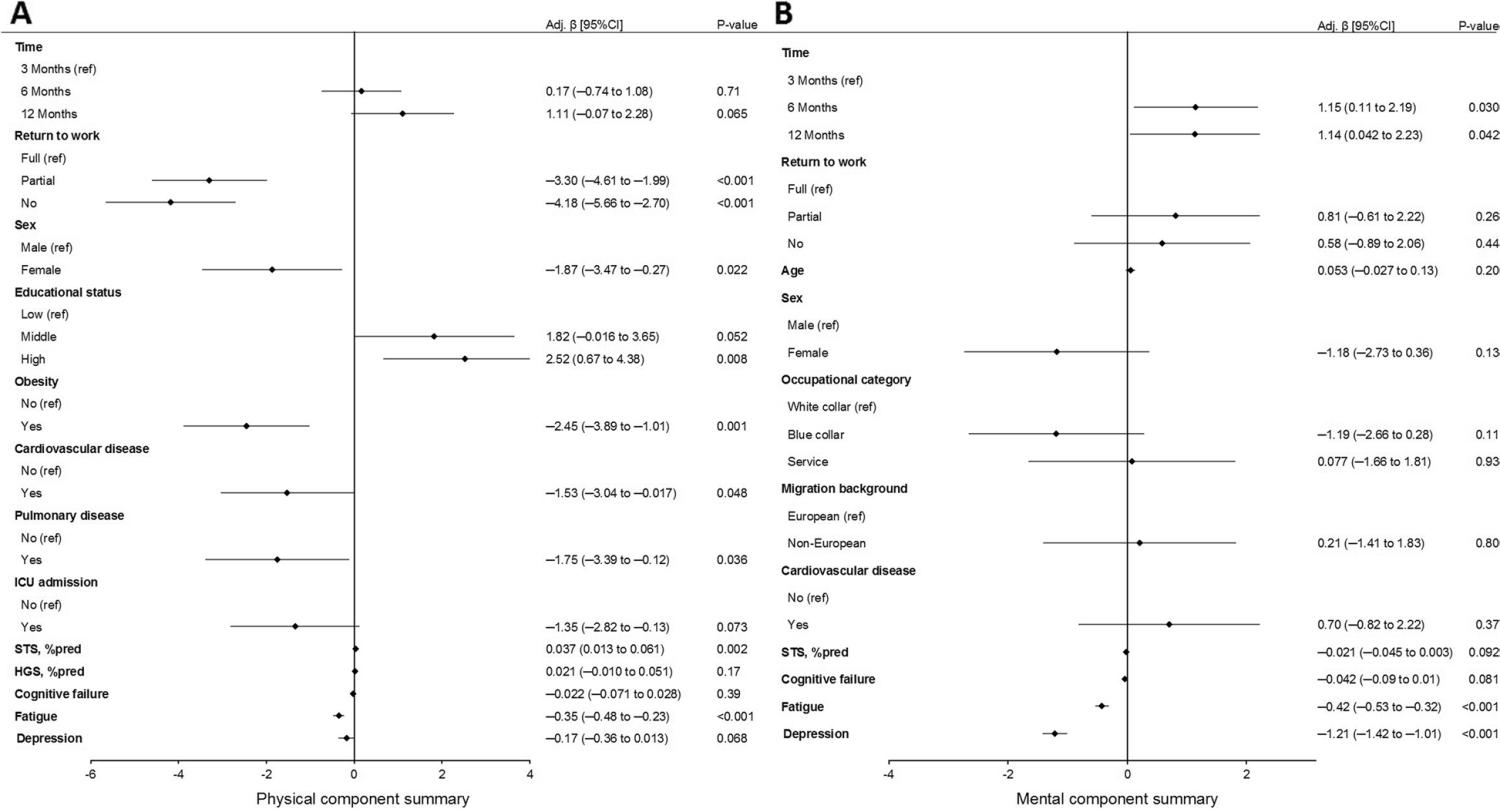
Forest plot showing adjusted odds ratios from multivariable analysis of **A** Physical Component Summary and **B** Mental Component Summary up to 1 year after hospitalization for COVID-19. ICU, intensive care unit; STS, sit-to-stand; %pred, percentage of normative values; HGS, handgrip strength

Univariable associations for PCS and MCS are presented in Additional file 1: Fig. S2. In multivariable analysis, return to work remained significantly associated with PCS after adjustment for covariables. In addition, female sex, lower educational status, history of obesity, cardiovascular disease, pulmonary disease, lower STS %pred, and persistent fatigue were negatively associated with PCS (Fig. 6A). Return to work was no longer significantly associated with MCS after adjustment for covariables, while persistent fatigue and depression were associated with a lower MCS (Fig. 6B).

## Discussion

Our study contributes to the limited body of literature and demonstrates the extent of not returning to work up to 1 year post-COVID-19, its risk factors, and its association with HRQoL. Overall, 50% of patients had not yet returned to work at 3 months after hospital discharge, decreasing to 29% at 6 months, and 15% at 12 months. At 12 months, another 16% of patients partially returned to work, resulting in only 69% fully returning to work. We found that differences in percentage in return to work at 3 months were no longer observed at 12 months between ICU and non-ICU patients, indicating that ICU patients required a longer time to return to work compared with non-ICU patients. Besides patients treated in ICU, those with fatigue, female sex, and older age were less likely to return to work. In addition to these factors, patients with a history of cardiovascular disease were less likely to fully return to work. We also showed that patients who did not return or partially returned to work had worse HRQoL in all domains at all follow-up moments compared with those who fully returned. Return to work was independently associated with PCS, but not with MCS.

The proportions of patients without ICU admission not returning to work, i.e., 33% and 19% at 3 and 6 months, respectively, align with other studies in hospitalized patients [9, 33]. Also, the proportions of those with ICU admission not returning to work at 3 and 6 months, 70% (107/153) and 43% (58/136), respectively, are similar to results of a Dutch post-ICU COVID-19 study (43%) [34], but somewhat higher than those of an Italian study (22%) [13], probably due to the small sample size of the latter or potential differences in ICU admission criteria, healthcare system, or labor market. Our study, along with others, has shown that ICU patients required more time to return to work compared with non-ICU patients [5, 9, 33]. Possibly, the post-intensive care syndrome (PICS), which refers to new or worsening physical, mental, and cognitive impairments in ICU survivors, complicates return to work [35]. A meta-analysis in PICS patients showed that 40% of patients did not return to work 12 months after ICU admission [36], similar to our findings in patients with COVID-19 who were treated in the ICU. A potential intervention to prevent PICS is early mobilization with physical and occupational therapy during ICU admission, which has also been shown to reduce the duration of delirium and length of ICU and hospital stay and mitigate long-term cognitive impairments [37–39]. To prevent PICS, which could in turn improve return to work and HRQoL, greater emphasis on rehabilitation during the early stages of ICU admission for COVID-19 could be recommended [36, 40].

Other effective interventions for improving return to work and HRQoL among individuals with chronic diseases and mental health conditions encompassed a multidisciplinary approach that addresses person-level components, such as symptom coping mechanisms, skills training, and goal establishment, and work-directed interventions involving adaption and evaluation of working task, schedule, and environment. These strategies, proven effective in previous contexts, hold promise for potential applicability among patients recovering from COVID-19 [41–44].

Other studies on patients hospitalized for COVID-19 and on non-COVID-19 severe acute respiratory syndrome survivors showed that 12–18% and 17%, respectively, of patients did not return to work at 12 months comparable to our findings [4, 45, 46]. We also found that 19% (48/251) of patients who returned to work at 12 months worked less hours than pre-hospitalization; Huang et al. reported an even higher proportion of 24% [4]. These findings suggest that COVID-19 could hinder patients’ ability to perform at pre-hospitalization levels for up to 12 months after discharge.

People who did not return or partially returned to work reported a significantly lower HRQoL compared with those who fully returned, which is in agreement with the literature [7, 13], although the direction of this association is unknown. Return to work was independently associated with PCS, but not with MCS. This could be due to the emphasis on work performance in the physical domains of the SF-36, which are more heavily weighed in PCS. Other factors independently associated with PCS were a history of obesity, cardiovascular and pulmonary disease, female sex, persistent fatigue, and lower physical fitness. MCS was associated with depression and persistent fatigue.

Persistent fatigue was independently associated with return to work and HRQoL, consistent with other studies [9, 47, 48], and reflecting the most common post-COVID-19 symptom [2, 49]. Post-COVID-19 fatigue is a multidimensional construct, affecting patients’ physical and mental health, and remains poorly understood [50, 51]. Post-COVID-19 fatigue is associated with shortness of breath, psychological distress, cognitive impairment, depression, and anxiety [52–54]. Treatment options, e.g., vocational rehabilitation programs that focus on energy management and pacing, could be beneficial to manage fatigue [55–57]. Moreover, physical rehabilitation could help improve physical fitness, and cognitive and psychological support could provide symptom relief. These interventions could promote a successful return to work and improve HRQoL. Overall, further research into post-COVID-19 fatigue is essential to develop effective treatment options.

Our study has some limitations. Isolation measures while recovering from COVID-19 and the anxiety for the consequences of COVID-19 could have had a negative impact on HRQoL. The data collection period coincided with two national lockdowns, which may have influenced HRQoL due to restrictions on social contact, travel, and leisure independent of previous infection; thus comparing with the Dutch norm of the SF-36 might not be appropriate. We did not collect data on changes in job responsibilities, limitations in work performance upon return to work, or the main reason for not returning to work. Job changes were not observed within 1 year, except for one patient. Moreover, we studied occupational categories, but did not find an effect on return to work. Lastly, although non-responders were younger, they were more frequently admitted to the ICU; therefore, it is hard to discern the effect on our results.

Strengths of our study include the recruitment of participants from academic and regional hospitals. This enabled us to include individuals with varying degrees of disease severity, which increases the external validity of the findings. Given the significant proportion of ICU patients, we were able to compare the ICU versus non-ICU patients within our cohort and with other ICU cohorts. Our findings may guide the development of rehabilitation programs tailored to the needs required for return to work. Additionally, the prospective design of our study with multiple follow-up time points enabled us to evaluate recovery up to 1 year. Finally, we used validated questionnaires to assess fatigue, cognitive, and psychological symptoms and objectively assessed the physical status. To our knowledge, this is the first comprehensive prospective cohort study to investigate the association between return to work and baseline characteristics and physical and mental recovery up to 1 year.

## Conclusions

All in all, 15% of patients did not return and 16% partially returned to work, leaving only 69% who fully returned to work 1 year after hospitalization for COVID-19. ICU admission, persistent fatigue, female sex, and older age were independently associated with return to work. Patients who did not return or partially returned to work reported lower HRQoL. More research is required to identify effective therapies for the long-term consequences of COVID-19, including vocational support and support for fatigue and physical, cognitive, and psychological symptoms.

### Supplementary Information

**Additional file 1: Table S1.** PROMs, physical outcomes, and HRQoL at 3, 6, and 12 months follow-up. **Table S2.** Estimated mean differences and 95% confidence interval of the SF-36 domains at 3, 6, and 12 months. **Table S3.** Estimated mean differences and 95% confidence interval of the SF-36 domains for no return to work versus partial or full return to work at 3, 6, and 12 months. **Table S4.** Scores on SF-36 domains for patients with no, partial, or full return to work at 3, 6, and 12 months after hospitalization for COVID-19. **Table S5.** Estimated mean differences and 95% confidence interval of the SF-36 domains for no or partial return to work versus full return to work at 3, 6, and 12 months. **Supplemental Fig. S1.** Forest plot showing odds ratios from univariable analysis of A] return to work (no versus partial/full) and B] full return to work (no/partial versus full) up to 1 year after hospitalization for COVID-19. ICU, Intensive Care Unit; M, months; LOS, Length Of Stay; 6MWD, 6 Min Walking Distance; %pred, percentage of normative values; STS, Sit-To-Stand; HGS, Handgrip Strength; PTSD, Posttraumatic Stress Disorder. **Supplemental Fig. S2.** Forest plot showing odds ratios from univariable analysis of A] Physical Component Summary and B] Mental Component Summary up to 1 year after hospitalization for COVID-19. ICU, Intensive Care Unit; LOS, Length Of Stay; 6MWD, 6 Min Walking Distance; %pred, percentage of normative values; STS, Sit-To-Stand; HGS, Handgrip Strength; PTSD, Posttraumatic Stress Disorder.

## Data Availability

The datasets used and/or analyzed during the current study are available from the corresponding author on reasonable request.

## Abbreviations

1MSTS: 1-Minute sit-to-stand test
6MWD: 6-Minute walking distance
6MWT: 6-Minute walk test
AOR: Adjusted odds ratios
BMI: Body mass index
BP: Bodily pain
CI: Confidence intervals
CO-FLOW: COvid-19 Follow-up care paths and Long-term Outcomes Within the Dutch healthcare system
COVID-19: Coronavirus disease 2019
GEE: Generalized estimating equations
GH: General health
HGS: Handgrip strength
ICU: Intensive care unit
iPCQ: iMTA Productivity cost questionnaire
IQR: Interquartile ranges
MCS: Mental component summary
MH: Mental health
PCS: Physical component summary
PF: Physical functioning
PICS: Post-intensive care syndrome
PROMs: Patient-reported outcome measures
PTSD: Posttraumatic stress disorder
RE: Role limitations due to emotional problems
RP: Role limitations due to physical health
SD: Standard deviation
SF: Social functioning
SF-36: Short form 36-item health survey
STS: Sit-to-stand
VT: Vitality
