# Supplementary material for: Return to work and health-related quality of life up to 1 year in patients hospitalized for COVID-19: the CO-FLOW study
Source: BMC Med. 2023 Oct 2;21:380. doi: 10.1186/s12916-023-03083-3 (PMC10546751; doi:10.1186/s12916-023-03083-3)
Supplement: Supplementary file 1 — Additional file 1: Table S1. PROMs, physical outcomes, and HRQoL at 3, 6, and 12 months follow-up. Table S2. Estimated mean differences and 95% confidence interval of the SF-36 domains at 3, 6, and 12 months. Table S3. Estimated mean differences and 95% confidence interval of the SF-36 domains for no return to work versus partial or full return to work at 3, 6, and 12 months. Table S4. Scores on SF-36 domains for patients with no, partial, or full return to work at 3, 6, and 12 months after hospitalization for COVID-19. Table S5. Estimated mean differences and 95% confidence interval of the SF-36 domains for no or partial return to work versus full return to work at 3, 6, and 12 months. Supplemental Fig. S1. Forest plot showing odds ratios from univariable analysis of A] return to work (no versus partial/full) and B] full return to work (no/partial versus full) up to 1 year after hospitalization for COVID-19. ICU, Intensive Care Unit; M, months; LOS, Length Of Stay; 6MWD, 6 Min Walking Distance; %pred, percentage of normative values; STS, Sit-To-Stand; HGS, Handgrip Strength; PTSD, Posttraumatic Stress Disorder. Supplemental Fig. S2. Forest plot showing odds ratios from univariable analysis of A] Physical Component Summary and B] Mental Component Summary up to 1 year after hospitalization for COVID-19. ICU, Intensive Care Unit; LOS, Length Of Stay; 6MWD, 6 Min Walking Distance; %pred, percentage of normative values; STS, Sit-To-Stand; HGS, Handgrip Strength; PTSD, Posttraumatic Stress Disorder. [file 12916_2023_3083_MOESM1_ESM.docx]

**Additional file 1**

**Return to work and health-related quality of life up to 1 year in patients hospitalized for COVID-19; the CO-FLOW study**

L.M. Bek, J.C. Berentschot, M.E. Hellemons, S.C. Remerie, J. van Bommel, J.G.J.V. Aerts, G.M. Ribbers, H.J.G. van den Berg-Emons ^*^, M.H. Heijenbrok-Kal^*^, on behalf of the CO-FLOW collaboration Group

**Contents:**

**Table S1**. PROMs, physical outcomes, and HRQoL at 3, 6, and 12 months follow-up.

**Table S2**. Estimated mean differences and 95% confidence interval of the SF-36 domains at 3, 6, and 12

months.

**Table S3**. Estimated mean differences and 95% confidence interval of the SF-36 domains for no return to work versus partial or full return to work at 3, 6, and 12 months.

**Table S4**. Scores on SF-36 domains for patients with no, partial, or full return to work at 3, 6, and 12 months after hospitalization for COVID-19.

**Table S5**. Estimated mean differences and 95% confidence interval of the SF-36 domains for no or partial return to work versus full return to work at 3, 6, and 12 months.

**Fig. S1.** Forest plot showing odds ratios from univariable analysis of A] return to work (no versus partial/full) and B] full return to work (no/partial versus full) up to 1 year after hospitalization for COVID-19.

**Fig. S2.** Forest plot showing odds ratios from univariable analysis of A] Physical Component Summary and B] Mental Component Summary up to 1 year after hospitalization for COVID-19.

| **Table S1.** PROMs, physical outcomes, and HRQoL at 3, 6, and 12 months follow-up. | | | | |
| --- | --- | --- | --- | --- |
|  | 3 Months | 6 Months | 12 Months | p-value ^c^ |
| **PROMs** |  |  |  |  |
| Cognitive failures ^a^ | 30.3 (1.1) | 31.1 (1.1) | 32.1 (1.1) | 0.280 |
| Fatigue ^a^ | 26.2 (0.5) | 24.7 (0.5) | 23.9 (0.5) | <0.001 |
| Anxiety ^a^ | 5.1 (0.2) | 4.8 (0.2) | 4.5 (0.2) | 0.003 |
| Depression ^a^ | 5.7 (0.2) | 5.1 (0.2) | 5.0 (0.2) | 0.003 |
| PTSD ^a^ | 15.4 (0.8) | 13.3 (0.8) | 12.7 (0.7) | <0.001 |
| **Physical outcomes** |  |  |  |  |
| 6MWD %pred ^b^ | 87.5 (1.2) | 90.4 (1.2) | 92.9 (1.1) | <0.001 |
| STS %pred ^b^ | 66.1 (1.6) | 74.3 (1.5) | 78.3 (1.7) | <0.001 |
| HGS %pred ^b^ | 92.5 (1.4) | 102.0 (1.3) | 108.3 (1.4) | <0.001 |
| **HRQoL** |  |  |  |  |
| n | 255 | 285 | 282 |  |
| Physical functioning | 65.6 (1.4) | 71.3 (1.3) | 75.4 (1.3) | <0.001 |
| Role limitation due to  physical health | 32.7 (2.5) | 44.9 (2.5) | 59.5 (2.5) | <0.001 |
| Role limitation due to  emotional problems | 58.4 (2.6) | 69.0 (2.4) | 73.4 (2.2) | <0.001 |
| Vitality | 53.0 (1.3) | 65.5 (1.2) | 58.9 (1.2) | <0.001 |
| Mental health | 73.4 (1.1) | 74.9 (1.0) | 76.4 (1.1) | 0.002 |
| Social functioning | 66.9 (1.8) | 74.3 (1.4) | 77.9 (1.4) | <0.001 |
| Bodily pain | 69.8 (1.5) | 73.5 (1.4) | 75.2 (1.4) | <0.001 |
| General health | 53.9 (1.2) | 65.1 (1.3) | 57.0 (1.3) | 0.006 |
| Physical component summary | 41.4 (0.6) | 43.8 (0.6) | 45.8 (0.6) | <0.001 |
| Mental component summary | 45.5 (0.7) | 47.5 (0.7) | 48.2 (0.6) | <0.001 |
| *Data are presented as estimated means with standard error, unless indicated. PROMs, Patient Reported Outcome Measures; PTSD, Posttraumatic Stress Disorder; 6MWD, 6 Min Walking Distance; %pred, percentage of normative values; STS, Sit-To-Stand; HGS, Handgrip Strength; HRQoL, Health-Related Quality of Life.*  *^a^ The number of patients who filled in the PROMs at 3, 6, and 12 months is as follows: Cognitive failures, n=264, 286, and 283; Fatigue, n=261, 287, and 282; Anxiety, n=258, 286, and 283; Depression, n=258, 286, and 284; PTSD, n=259, 287, and 286, respectively.*  *^b^ The number of patients assessed on the physical outcomes at 3, 6, and 12 months is as follows: 6MWT, n=237, 254, and 288; 1MSTS, n=290, 304, and 311; HGS, n=266, 283, and 288, respectively.*  *^c^ p-values are obtained with linear generalized estimating equations with repeated measurements.* | | | | |

**Table S2.** Estimated mean differences and 95% confidence interval of the SF-36 domains at 3, 6, and 12 months.

|  | **Follow-up time** | | |
| --- | --- | --- | --- |
| **SF-36 domains** | 3 Months | 6 Months | 12 Months |
| Physical functioning | Ref. | 5.8 (3.9 to 7.6)  p<0.001 | 9.9 (7.6 to 12.2)  p<0.001 |
| Role limitation due to  physical health | Ref. | 12.2 (7.3 to 17.1)  p<0.001 | 26.8 (21.5 to 32.1)  p<0.001 |
| Role limitation due to emotional problems | Ref. | 10.6 (5.8 to 15.3)  p<0.001 | 15.0 (9.9 to 20.2)  p<0.001 |
| Vitality | Ref. | 3.5 (1.5 to 5.5)  p<0.001 | 5.9 (3.7 to 8.2)  p<0.001 |
| Mental health | Ref. | 1.5 (0.4 to 3.1)  p=0.05 | 3.0 (1.4 to 4.7)  p<0.001 |
| Social functioning | Ref. | 7.4 (4.4 to 10.4)  p<0.001 | 11.1 (7.9 to 14.3)  p<0.001 |
| Bodily pain | Ref. | 3.7 (1.4 to 6.0)  p=0.002 | 5.4 (2.8 to 8.0)  p<0.001 |
| General health | Ref. | 2.2 (0.4 to 5.7)  p=0.017 | 3.1 (1.1 to 5.0)  p=0.002 |
| Physical component summary | Ref. | 2.3 (1.5 to 3.1)  p<0.001 | 4.4 (3.4 to 5.3)  p<0.001 |
| Mental component summary | Ref. | 2.0 (1.0 to 3.1)  p<0.001 | 2.7 (1.6 to 3.9)  p<0.001 |

Estimated mean differences are obtained using linear generalized estimating equations.

**Table S3.** Estimated mean differences and 95% confidence interval of the SF-36 domains for no return to work versus partial or full return to work at 3, 6, and 12 months.

|  | **Follow-up time** | | |
| --- | --- | --- | --- |
| **SF-36 domains** | 3 Months | 6 Months | 12 Months |
| **Physical functioning** |  |  |  |
| No | Ref. | Ref. | Ref. |
| Partial or full | 7.4 (3.4 to 11.5)  p<0.001 | 10.1 (5.7 to 14.4) p<0.001 | 10.4 (2.4 to 18.4) p=0.011 |
| **Role limitation due to**  **physical health** |  |  |  |
| No | Ref. | Ref. | Ref. |
| Partial or full | 17.8 (9.0 to 26.7) p<0.001 | 28.6 (20.0 to 37.1) p<0.001 | 40.9 (29.8 to 52.0) p<0.001 |
| **Role limitation due to emotional problems** |  |  |  |
| No | Ref. | Ref. | Ref. |
| Partial or full | 8.7 (─0.7 to 18.0)  p=0.069 | 16.7 (5.9 to 27.6) p=0.002 | 24.6 (12.2 to 36.9) p<0.001 |
| **Vitality** |  |  |  |
| No | Ref. | Ref. | Ref. |
| Partial or full | 4.2 (0.2 to 8.3)  p=0.038 | 3.1 (─0.7 to 7.0)  p=0.108 | 5.1 (─0.3 to 10.6)  p=0.066 |
| **Mental health** |  |  |  |
| No | Ref. | Ref. | Ref. |
| Partial or full | 3.6 (0.5 to 6.8)  p=0.024 | 3.9 (0.6 to 7.2)  p=0.021 | 3.8 (─0.3 to 8.0)  p=0.071 |
| **Social functioning** |  |  |  |
| No | Ref. | Ref. | Ref. |
| Partial or full | 11.7 (5.7 to 17.8)  p<0.001 | 11.6 (5.7 to 17.5)  p<0.001 | 14.8 (7.0 to 22.6)  p<0.001 |
| **Bodily pain** |  |  |  |
| No | Ref. | Ref. | Ref. |
| Partial or full | 6.5 (2.0 to 10.9)  p=0.005 | 9.2 (4.1 to 14.2)  p<0.001 | 14.8 (7.3 to 22.3)  p<0.001 |
| **General health** |  |  |  |
| No | Ref. | Ref. | Ref. |
| Partial or full | 4.6 (1.1 to 8.0)  p=0.009 | 4.1 (─0.1 to 8.3)  p=0.054 | 5.4 (─0.8 to 11.5)  p=0.089 |
| **Physical Component Summary** |  |  |  |
| No | Ref. | Ref. | Ref. |
| Partial or full | 2.8 (1.2 to 4.4)  p<0.001 | 4.3 (2.6 to 6.0)  p<0.001 | 7.0 (4.3 to 9.6)  p<0.001 |
| **Mental Component Summary** |  |  |  |
| No | Ref. | Ref. | Ref. |
| Partial or full | 2.1 (0.1 to 4.2)  p=0.044 | 2.1 (─0.3 to 4.4)  p=0.087 | 2.1 (─0.8 to 5.1)  p=0.158 |

Estimated mean differences are obtained using linear generalized estimating equations.

| **Table S4**. Scores on SF-36 domains for patients with no, partial, or full return to work at 3, 6, and 12 months after hospitalization for COVID-19. | | | | | | |
| --- | --- | --- | --- | --- | --- | --- |
| SF-36 domains | Dutch norm | No return to work | Partial return to work | Full return to work | p-value ^a^ | p-value ^b^ |
| **3 Months** | | | | | | |
| n (%) |  | 114 (48) | 53 (22) | 73 (30) |  |  |
| Physical functioning | 83.0 | 60.7 (1.9) | 64.4 (2.3) | 74.2 (1.8) | <0.001 | <0.001 |
| Role limitation due to  physical health | 76.4 | 19.7 (2.8) | 25.9 (5.0) | 57.4 (4.7) | <0.001 | <0.001 |
| Role limitation due to  emotional problems | 82.3 | 53.0 (3.7) | 52.7 (5.2) | 72.5 (4.1) | 0.069 | <0.001 |
| Vitality | 68.6 | 50.1 (1.7) | 50.9 (2.4) | 58.8 (1.9) | 0.038 | <0.001 |
| Mental health | 76.8 | 71.1 (1.5) | 71.8 (1.8) | 78.1 (1.5) | 0.024 | <0.001 |
| Social functioning | 84.0 | 59.7 (2.6) | 66.8 (3.4) | 78.0 (2.4) | <0.001 | <0.001 |
| Bodily pain | 74.9 | 65.5 (2.0) | 69.1 (2.5) | 78.0 (2.1) | 0.005 | <0.001 |
| General health | 70.7 | 51.0 (1.7) | 53.8 (1.8) | 58.3 (1.7) | 0.009 | <0.001 |
| Physical component summary | 50.0 | 39.3 (0.7) | 41.2 (1.0) | 45.1 (0.8) | <0.001 | <0.001 |
| Mental component summary | 50.0 | 44.3 (0.9) | 44.4 (1.3) | 48.5 (1.0) | 0.044 | <0.001 |
| **6 Months** | | | | | | |
| n (%) |  | 70 (26) | 58 (22) | 137 (52) |  |  |
| Physical functioning | 83.0 | 62.6 (2.2) | 67.8 (2.1) | 76.5 (1.3) | <0.001 | <0.001 |
| Role limitation due to  physical health | 76.4 | 18.2 (3.4) | 26.9 (4.1) | 64.8 (3.1) | <0.001 | <0.001 |
| Role limitation due to  emotional problems | 82.3 | 55.2 (5.1) | 64.8 (4.7) | 76.9 (2.8) | 0.002 | <0.001 |
| Vitality | 68.6 | 53.0 (1.9) | 54.0 (2.0) | 58.6 (1.5) | 0.108 | 0.004 |
| Mental health | 76.8 | 71.2 (1.7) | 73.1 (1.8) | 77.2 (1.1) | 0.021 | <0.001 |
| Social functioning | 84.0 | 64.6 (2.8) | 70.3 (2.6) | 80.7 (1.6) | <0.001 | <0.001 |
| Bodily pain | 74.9 | 65.4 (2.6) | 68.2 (2.4) | 79.1 (1.4) | <0.001 | <0.001 |
| General health | 70.7 | 52.4 (2.2) | 54.7 (1.9) | 58.6 (1.5) | 0.054 | 0.006 |
| Physical component summary | 50.0 | 39.8 (0.9) | 41.2 (0.8) | 46.6 (0.6) | <0.001 | <0.001 |
| Mental component summary | 50.0 | 45.7 (1.2) | 47.0 (1.3) | 48.6 (0.7) | 0.087 | 0.027 |
| **12 Months** | | | | | | |
| n (%) |  | 40 (15) | 44 (17) | 176 (68) |  |  |
| Physical functioning | 83.0 | 64.8 (4.0) | 67.4 (2.8) | 78.9 (1.3) | 0.011 | <0.001 |
| Role limitation due to  physical health | 76.4 | 20.0 (4.8) | 34.4 (5.1) | 73.3 (2.6) | <0.001 | <0.001 |
| Role limitation due to  emotional problems | 82.3 | 50.7 (6.0) | 59.9 (5.9) | 80.7 (2.2) | <0.001 | <0.001 |
| Vitality | 68.6 | 53.2 (2.7) | 54.3 (2.2) | 60.3 (1.4) | 0.066 | 0.002 |
| Mental health | 76.8 | 72.1 (2.2) | 72.2 (2.0) | 77.6 (1.2) | 0.071 | <0.001 |
| Social functioning | 84.0 | 63.8 (3.8) | 71.0 (3.6) | 82.0 (1.3) | <0.001 | <0.001 |
| Bodily pain | 74.9 | 61.7 (3.8) | 66.5 (2.9) | 80.5 (1.4) | <0.001 | <0.001 |
| General health | 70.7 | 51.2 (3.0) | 52.9 (1.9) | 58.2 (1.4) | 0.089 | 0.004 |
| Physical component summary | 50.0 | 39.0 (1.3) | 41.5 (1.1) | 48.2 (0.6) | <0.001 | <0.001 |
| Mental component summary | 50.0 | 46.0 (1.5) | 46.0 (1.2) | 48.9 (0.7) | 0.158 | 0.009 |
| *Data are presented as estimated mean with standard error, unless indicated. p-values are obtained with linear generalized estimating equations with repeated measurements.*  *^a^ difference between no return to work and full/partial return to work, ^b^ difference between no/partial return to work and full return to work. SF-36, 36-Item Short Form Health Survey* | | | | | | |

**Table S5.** Estimated mean differences and 95% confidence interval of the SF-36 domains for no or partial return to work versus full return to work at 3, 6, and 12 months.

|  | **Follow-up time** | | |
| --- | --- | --- | --- |
| **SF-36 domains** | 3 Months | 6 Months | 12 Months |
| **Physical functioning** |  |  |  |
| No or partial | Ref. | Ref. | Ref. |
| Full | 12.3 (8.3 to 16.3)  p<0.001 | 10.9 (7.4 to 14.3)  p<0.001 | 12.1 (7.2 to 17.0)  p<0.001 |
| **Role limitation due to**  **physical health** |  |  |  |
| No or partial | Ref. | Ref. | Ref. |
| Full | 35.8 (25.4 to 46.2)  p<0.001 | 42.1 (34.2 to 49.9)  p<0.001 | 45.2 (36.7 to 53.7)  p<0.001 |
| **Role limitation due to emotional problems** |  |  |  |
| No or partial | Ref. | Ref. | Ref. |
| Full | 19.9 (10.2 to 29.7)  p<0.001 | 17.4 (8.7 to 26.1)  p<0.001 | 24.8 (15.5 to 34.1)  p<0.001 |
| **Vitality** |  |  |  |
| No or partial | Ref. | Ref. | Ref. |
| Full | 8.6 (4.3 to 12.9)  p<0.001 | 5.3 (1.7 to 8.9)  p=0.004 | 6.9 (2.9 to 10.9)  p<0.001 |
| **Mental health** |  |  |  |
| No or partial | Ref. | Ref. | Ref. |
| Full | 6.7 (3.6 to 9.9)  p<0.001 | 5.0 (2.1 to 7.8)  p<0.001 | 5.3 (2.1 to 8.5)  p<0.001 |
| **Social functioning** |  |  |  |
| No or partial | Ref. | Ref. | Ref. |
| Full | 16.1 (10.3 to 22.0)  p<0.001 | 12.9 (8.1 to 17.6)  p<0.001 | 13.7 (8.0 to 19.3)  p<0.001 |
| **Bodily pain** |  |  |  |
| No or partial | Ref. | Ref. | Ref. |
| Full | 11.3 (6.5 to 16.1)  p<0.001 | 12.0 (7.9 to 16.0)  p<0.001 | 15.6 (10.4 to 20.7)  p<0.001 |
| **General health** |  |  |  |
| No or partial | Ref. | Ref. | Ref. |
| Full | 6.5 (2.9 to 10.0)  p<0.001 | 5.0 (1.4 to 8.6)  p=0.006 | 5.8 (1.8 to 9.7)  p=0.004 |
| **Physical Component Summary** |  |  |  |
| No or partial | Ref. | Ref. | Ref. |
| Full | 5.1 (3.2 to 7.0)  p<0.001 | 5.9 (4.3 to 7.4)  p<0.001 | 7.7 (5.8 to 9.6)  p<0.001 |
| **Mental Component Summary** |  |  |  |
| No or partial | Ref. | Ref. | Ref. |
| Full | 4.2 (2.0 to 6.4)  p<0.001 | 2.3 (0.3 to 4.3)  p=0.027 | 2.8 (0.7 to 5.0)  p=0.009 |

Estimated mean differences are obtained using linear generalized estimating equations.


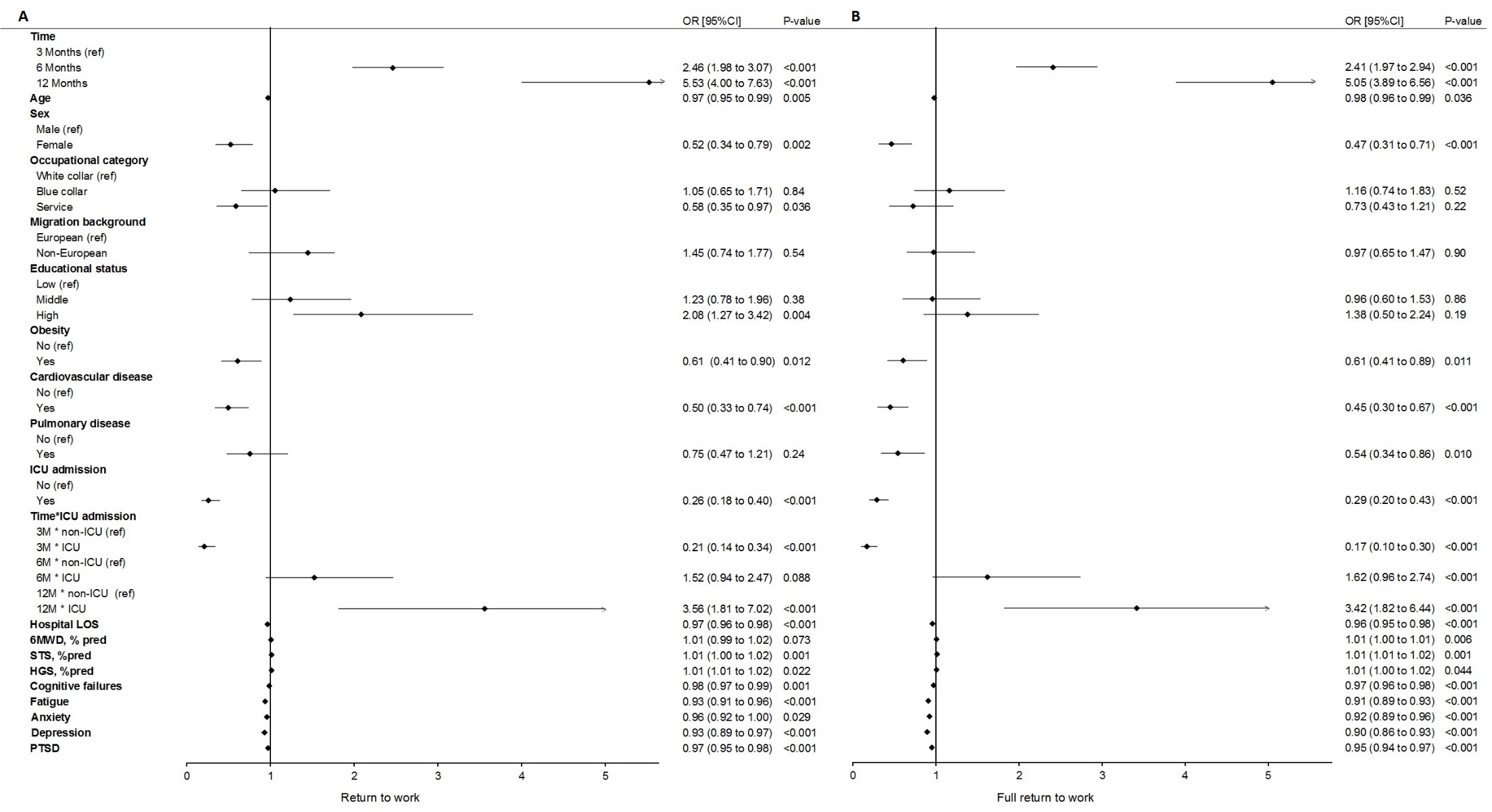
**Fig. S1.** Forest plot showing odds ratios from univariable analysis of A] return to work (no versus partial/full) and B] full return to work (no/partial versus full) up to 1 year after hospitalization for COVID-19. ICU, Intensive Care Unit; M, months; LOS, Length Of Stay; 6MWD, 6 Min Walking Distance; %pred, percentage of normative values; STS, Sit-To-Stand; HGS, Handgrip Strength; PTSD, Posttraumatic Stress Disorder.


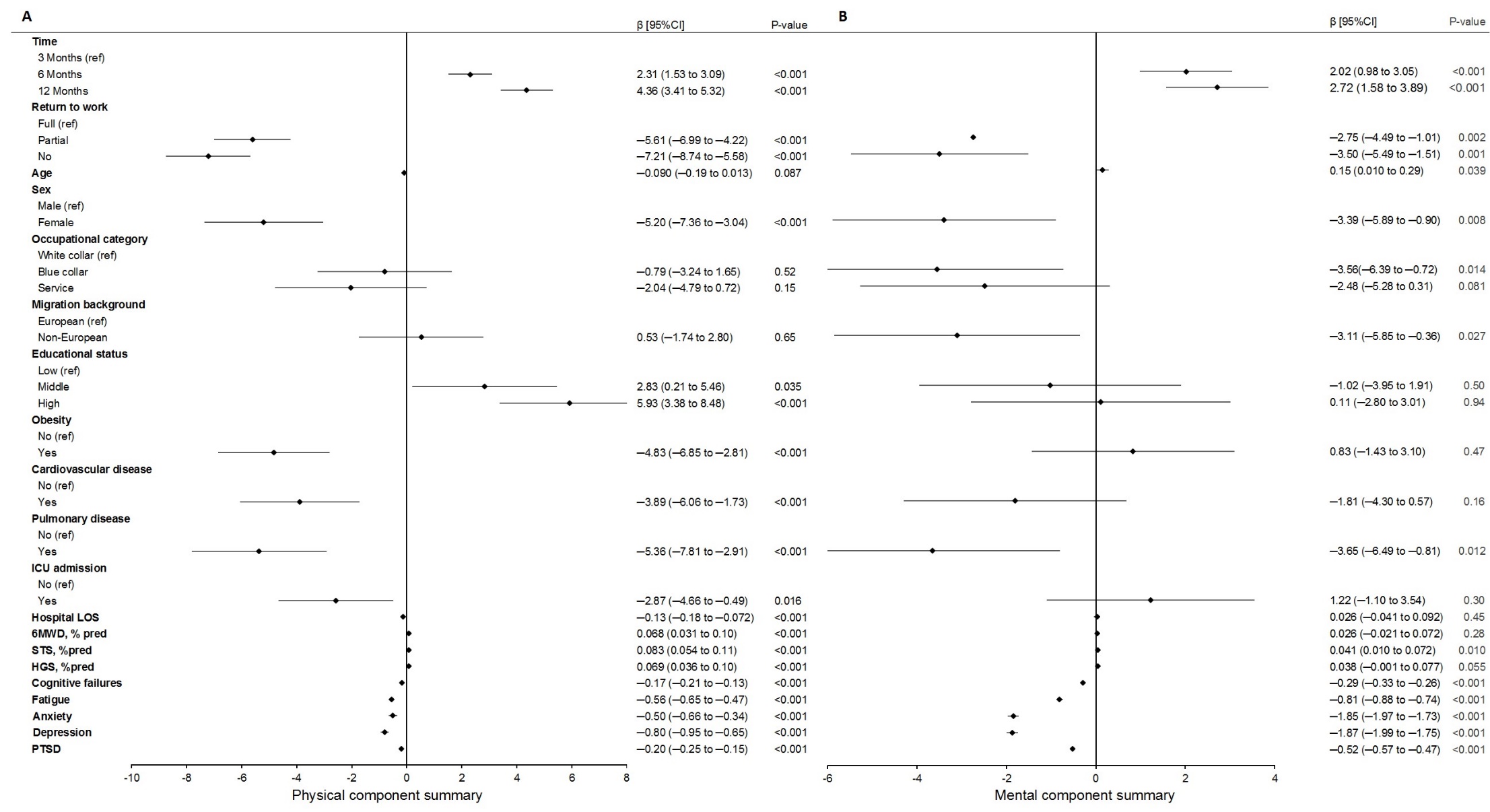
**Fig. S2.** Forest plot showing odds ratios from univariable analysis of A] Physical Component Summary and B] Mental Component Summary up to 1 year after hospitalization for COVID-19. ICU, Intensive Care Unit; LOS, Length Of Stay; 6MWD, 6 Min Walking Distance; %pred, percentage of normative values; STS, Sit-To-Stand; HGS, Handgrip Strength; PTSD, Posttraumatic Stress Disorder.
